# Tracing the Itch: A Spatiotemporal Analysis of Scabies Rates and Its Risk Factors Using the Global Burden of Disease 2021 Data

**DOI:** 10.1111/tmi.70029

**Published:** 2025-09-13

**Authors:** Saptorshi Gupta, Simon Thornley, Arthur Morris, Gerhard Sundborn, Cameron Grant

**Affiliations:** ^1^ Section of Biostatistics and Epidemiology, Faculty of Medical and Health Sciences The University of Auckland Auckland New Zealand; ^2^ LabPlus, Auckland City Hospital Auckland New Zealand; ^3^ Section of Pacific Health, Faculty of Medical and Health Sciences The University of Auckland Auckland New Zealand; ^4^ Department of Pediatrics, Child and Youth Health, Faculty of Medical and Health Sciences The University of Auckland Auckland New Zealand

**Keywords:** incidence, prevalence, risk factors, scabies, trends

## Abstract

**Background and Objectives:**

Scabies is a neglected disease believed to be more prevalent in resource‐poor nations. Published data describing global trends in scabies incidence and prevalence rates and factors associated with global regional differences are limited. Identifying regions with scabies prevalence rates over 10% and implementing mass‐drug administration is recommended. We aimed to identify global high‐risk areas to facilitate region‐specific targeted interventions.

**Methods:**

Data on scabies incidence and prevalence in 204 countries and regions from 1990 to 2021 were extracted from the Global Burden of Disease database. Temporal trends in age‐standardised rates were estimated using Joinpoint regression. Local indicators of spatial association were used to determine contiguous areas of high prevalence. The association of socio‐demographic and economic factors with scabies was determined using locally weighted scatterplot smoothing and log‐normal regression models.

**Results:**

Global prevalence of scabies in 2021 was 2.71% (95% confidence interval [CI]: 2.41% to 3.04%). Age‐standardised rates of scabies have marginally declined globally from 1990 to 2021 with an Average Annual Percentage Change (AAPC) of −0.10 (95% CI: −0.05 to −0.14) for incidence and −0.09 (95% CI: −0.05 to −0.14) for prevalence. Spatial clustering of high scabies prevalence was present in tropical Latin America, Southeast Asia, and the Pacific Islands. Rates have shown an increasing trend over time in high‐income regions such as Australasia and parts of Europe. Scabies rates have increased over time in high‐middle and high sociodemographic index regions. There is a significant positive association between warmer latitudes with increasing urbanisation and scabies prevalence.

**Conclusion:**

Owing to the exploratory nature of the GBD data, our findings are hypothesis generating, rather than confirmatory. Scabies prevalence remains high in several global regions. Progress to reduce scabies prevalence is slow with existing programmes. Scabies control policies should be further prioritised to accelerate progress in reducing the prevalence of this important tropical disease.

## 1 Introduction

Caused by *Sarcoptes scabiei* var. *hominis*, human scabies is an infectious disease that presents with symptoms of itch and rash, with secondary bacterial infections [1] or impetigo, causing frequent complications [2]. The need to relieve itch often leads to excoriation [1], which creates opportunity for infections with *Streptococcus pyogenes* , in turn resulting in further complications including post‐streptococcal glomerulonephritis [3], acute rheumatic fever (ARF) and rheumatic heart disease (RHD) [4, 5]. Since the health and economic burden of scabies is substantial, it is important to understand its distribution and determinants at global, national, and regional levels.

The World Health Organization (WHO) estimates the total global count of scabies cases to be at 200 million at any given time [6]. Scabies prevalence is disproportionately higher in tropical regions, especially among children, the elderly and the immunocompromised [1]. Despite its considerable health impacts and high prevalence, scabies remains a relatively neglected disease with mass‐drug administration (MDA) programs implemented often sub‐nationally [7], limited only to high‐risk areas, rather than being co‐ordinated regionally. Prioritising scabies as an emerging disease, through improved diagnosis and frequent screening, and conducting more comprehensive MDA programs, could further improve national control.

Understanding the distribution and determinants of scabies and its changing temporal trends could help guide disease control policy. Published estimates of spatiotemporal trends of scabies at the global, national and regional levels and risk factors for scabies are limited. This study uses data from the Global Burden of Diseases (GBD) study to derive these estimates from 1990 to 2021 and identify the high‐risk areas where scabies prevalence is increasing.

## 2 Methods

### 2.1 GBD Study

The GBD study is a detailed and comprehensive data collection that estimates 459 risk factors and health conditions both globally and nationally [8]. Estimates of incidence, prevalence, mortality, years of life lost (YLLs), years of life lived with disability (YLDs) and disability‐adjusted life years (DALYs) are reported for each condition.

For this study, the incidence, prevalence and age‐standardised rates of incidence (ASIR) and prevalence (ASPR) for scabies were obtained by sex and age group for 21 regions, 204 countries or territories for 1990 to 2021 from the online GHDx data source query tool (http://www.healthdata.org/gbd/), where the details of GBD data collection are described. In case of missing values, data from neighbouring areas were used as estimates to impute data [9].

Countries were categorised based on region, income, and sociodemographic index (SDI)—a composite index of development based on lagged per capita income, average level of education for populations aged 15 years and above, and total fertility rates for women less than 25 years old. Sociodemographic index values range from 0 (poorest) to 1 (wealthiest) with this index used to evaluate the development status of each location year. Countries were classified into five development quintiles based on the 2021 GBD data: low, low‐middle, middle, high‐middle, and high SDI regions [8].

### 2.2 Scabies Definition

The definition of scabies was based on the International Classification of Diseases (ICD) diagnostic criteria. Scabies cases were identified from ICD‐10 coded B86 cases from outpatient clinic data and a comprehensive literature review which included surveys or other published estimates of the frequency of disease.

### 2.3 Data Sources

For the GBD data collection, scabies data was extracted from a comprehensive literature review of previously published studies, USA claims data, outpatient data and USA MarketScan 2000 data. There has been an important change in modelling and data sources available compared to past GBD estimates. USA claims data from 2000 to 2010 through 2016 and outpatient data were included and for modelling, within‐DisMod crosswalks were replaced with crosswalks completed using the MR‐BRT modelling tool [10].

Five covariates—urban population as a percentage of total population, population density (number of people per square kilometre of land area), life expectancy at birth (LEB), unemployment as a percentage of total labour force, and Gross National Income (GNI) per capita by purchasing power parity (PPP) 2021 were collected from the DataBank of the World Bank's World Development Indicators [11].

### 2.4 Statistical Analyses

To facilitate comparison among several countries, age‐standardised rates (ASR) of scabies incidence and prevalence were directly collected from the GBD database and analysed using R [12] and QGis [13]. In the GBD analysis, ‘incidence’ refers to the number of new count cases during a specific time period in a particular population, the rates measured by the number of new cases in a year divided by the mid‐year population. ‘Prevalence’, on the other hand, refers to the total number of scabies cases in a specified population at a designated time point. The GBD study reports only ‘point prevalence’ and thus all estimates in this paper refer to this metric, indicating the number of people with scabies at a particular point in time. More specifically, prevalences are reported as an aggregated point estimate for a particular year. We have also used ‘age‐standardised prevalence’ (ASPR) and incidence rates of scabies in our analysis. This is to make the scabies burden comparable for between‐country analyses. The ASPR refers to the prevalence of a disease or condition per 100,000 population, adjusted to remove the influence of differences in age structure between populations being compared. Temporal changes in rates were calculated using Joinpoint regression software [14]. Trends were determined by calculating the ‘average percentage change’ (APC) and ‘annual average percentage change’ (AAPC), a summary widely used to measure ASR trends. The APC was calculated by taking the geometric mean of scabies rates for each year and fitting a straight line with this geometric mean as the dependent variable and year as the independent variable.

$${\mathrm{APC}}_{j}=100\times \left(e^{\beta_{j}}-1\right);\text{AAPC}=100\times \left(e^{\frac{\sum_{j=1}^{m}w_{j}\beta_{j}}{\sum_{j=1}^{m}w_{j}}}-1\right)$$

where $\beta_{j}:\text{slope of the}\;j\mathrm{th}\;\text{time segments};w_{j}:\text{is the length of the}\;j\mathrm{th}\;\text{time segment}$. A minimum of 0 and maximum of 6 join points were selected using the ‘grid search’ method over the study period. The permutation test was used for modelling. A parametric method was used to calculate the confidence intervals for APC and AAPC. A two‐sided *p*‐value of less than 0.05 indicated that APC and AAPC were significantly different from the null value (zero), representing no change. Trends are considered increasing when both AAPC and ASR are greater than zero and decreasing when both are less than zero.

The association of temporal trends in prevalence rates in major regions and socio‐demographic index (SDI) was determined using locally weighted scatterplot smoothing (LOWESS). Global Moran's *I* and local indicators of spatial association (LISA) or Local Moran's *I* were used to detect clusters of high scabies prevalence between neighbouring countries. Moran's *I* ranges from −1 (perfect dispersion) to 1 (perfect clustering).

A log‐linear regression model was used to determine the association between scabies rates and socioeconomic and geographic factors. Absolute values of latitude were taken to consider the equator as a baseline and latitude on either side as equal. Right skewed data was log‐transformed to restore normality, and multicollinearity between covariates was assessed before performing the regression analysis. A variance inflation factor (VIF) of more than 10 was considered substantial evidence of multicollinearity.

A conceptual framework of the data collection process and statistical analysis has been enumerated in Figure S1.

## 3 Results

### 3.1 Global Distribution of Scabies Burden

Scabies accounted for about 153 million cases (95% CI: 134 million to 174 million) in 1990 and 206 million cases (95% CI: 184 million to 231 million) in 2021. The ASPR decreased significantly from 2.74 per 100 (95% CI: 2.43 to 3.08) in 1990 to 2.67 per 100 (95% CI: 2.37 to 2.99) in 2021, with an AAPC of −0.09 (95% CI: −0.05 to −0.14).

Globally, the total cases of scabies incidence increased by 34.6% for both sexes from 462 million cases (95% CI: 407 million to 526 million) in 1990 to 622 million (95% CI: 556 million to 694 million) in 2021 (Table 1). However, the age‐standardised incidence rate (ASIR) of scabies showed a significant decrease of 2.7% over this period from 8.27 per 100 (95% CI: 7.32 to 9.31) in 1990 to 8.05 per 100 (95% CI: 7.17 to 9.02) in 2021, with an average annual percentage change (AAPC) of −0.10 (95% CI: −0.05 to −0.14).

**TABLE 1:** Incidence and prevalence of scabies and its rate of change from 1990 to 2019 in major parts of the world.

Characteristics	Incidence					Prevalence				
	1990		2021		AAPC (95% CI)	1990		2021		AAPC (95% CI)
	Cases × 10^3^ (95% CI)	ASIR per 100	Cases × 10^3^ (95% CI)	ASIR per 100		Cases × 10^3^ (95% CI)	ASPR per 100	Cases × 10^3^ (95% CI)	ASPR per 100	
Global	462,794 (407,240 to 526,071)	8.27 (7.32 to 9.31)	622,474 (556,235 to 694,992)	8.05 (7.17 to 9.02)	−0.10 (−0.05 to −0.14)	153,020 (134,417 to 174,050)	2.74 (2.43 to 3.08)	206,550 (184,176 to 231,741)	2.67 (2.37 to 2.99)	−0.09 (−0.05 to −0.14)
GBD Region										
Africa	26,970 (24,151 to 30,542)	3.95 (3.58 to 4.37)	54,560 (47,836 to 62,469)	3.94 (3.46 to 4.52)	−0.20 (−0.25 to −0.14)	9005 (8037 to 10,084)	1.33 (1.20 to 1.47)	18,158 (15,847 to 20,730)	1.24 (1.10 to 1.40)	−0.22 (−0.28 to −0.16)
America	54,256 (47,856 to 61,799)	7.38 (6.54 to 8.36)	76,545 (68,716 to 85,566)	8.09 (7.19 to 9.14)	0.30 (0.26 to 0.34)	18,030 (15,834 to 20,576)	2.45 (2.17 to 2.78)	25,450 (22,684 to 28,521)	2.68 (2.38 to 3.03)	0.29 (0.26 to 0.33)
Asia	371,238 (326,529 to 421,656)	11.14 (9.88 to 12.48)	481,288 (429,682 to 536,796)	10.76 (9.56 to 12.09)	−0.12 (−0.17 to −0.07)	122,551 (107,076 to 139,886)	3.67 (3.26 to 4.15)	159,585 (142,005 to 179,444)	3.56 (3.15 to 4.01)	−0.12 (−0.17 to −0.06)
Europe	9406 (8385 to 10,593)	1.23 (1.09 to 1.39)	9114 (8186 to 10,127)	1.20 (1.07 to 1.36)	−0.08 (−0.09 to −0.07)	3128 (2779 to 3529)	0.41 (0.36 to 0.46)	3035 (2723 to 3399)	0.40 (0.35 to 0.45)	−0.07 (−0.08 to −0.07)
Andean Latin America	4635 (4034 to 5928)	11.18 (9.88 to 12.59)	7314 (6474 to 8228)	11.19 (9.92 to 12.52)	0.00 (0.00 to 0.00)	1536 (1330 to 1771)	3.71 (3.27 to 4.21)	2431 (2141 to 2761)	3.71 (3.27 to 4.21)	0.001 (0.00 to 0.003)
Australasia	73 (64 to 83)	3.77 (3.31 to 4.32)	104 (92 to 115)	3.79 (3.33 to 4.31)	0.04 (−0.02 to 0.11)	24 (21 to 28)	1.26 (1.09 to 1.44)	35 (31 to 39)	1.26 (1.10 to 1.44)	0.05 (0.01 to 0.09)
Caribbean	4100 (3595 to 4622)	11.19 (9.90 to 12.52)	5128 (4564 to 5739)	11.21 (9.92 to 12.58)	0.00 (−0.01 to 0.02)	1360 (1188 to 1553)	3.72 (3.28 to 4.21)	1705 (1513 to 1914)	3.72 (3.28 to 4.21)	0.003 (−0.01 to 0.02)
Central Asia	1266 (1113 to 1459)	1.74 (1.55 to 1.98)	1654 (1469 to 1870)	1.74 (1.54 to 1.97)	0.00 (0.00 to 0.00)	417 (363 to 479)	0.58 (0.51 to 0.65)	547 (483 to 618)	0.58 (0.51 to 0.65)	−0.001 (−0.003 to 0.002)
Central Europe	5342 (4766 to 5992)	4.38 (3.89 to 4.92)	4722 (4225 to 5272)	4.41 (3.94 to 4.98)	0.03 (0.02 to 0.03)	1778 (1590 to 2010)	1.46 (1.29 to 1.64)	1574 (1414 to 1752)	1.47 (1.31 to 1.65)	0.03 (0.03 to 0.03)
Central Latin America	16,633 (14,461 to 19,117)	9.15 (8.14 to 10.33)	24,585 (21,794 to 27,640)	9.97 (8.83 to 11.24)	0.28 (0.26 to 0.30)	5491 (4758 to 6270)	3.03 (2.67 to 3.41)	8167 (7203 to 9253)	3.30 (2.91 to 3.73)	0.28 (0.27 to 0.30)
Central Sub‐Saharan Africa	1700 (1499 to 1958)	2.94 (2.64 to 3.31)	4223 (3700 to 4819)	2.95 (2.64 to 3.32)	0.01 (0.00 to 0.02)	556 (484 to 633)	0.97 (0.86 to 1.09)	1388 (1209 to 1584)	0.98 (0.87 to 1.10)	0.01 (0.001 to 0.02)
East Asia	181,500 (159,972 to 205,610)	14.54 (12.98 to 16.23)	198,513 (177,211 to 220,994)	14.56 (12.97 to 16.30)	0.00 (0.00 to 0.01)	59,995 (52,678 to 68,210)	4.80 (4.27 to 5.38)	65,861 (58,746 to 73,336)	4.81 (4.28 to 5.40)	0.003 (−0.004 to 0.01)
Eastern Europe	1923 (1718 to 2152)	0.89 (0.79 to 1.01)	1681 (1508 to 1867)	0.92 (0.82 to 1.05)	0.11 (0.10 to 0.11)	643 (573 to 723)	0.30 (0.26 to 0.34)	562 (502 to 627)	0.31 (0.28 to 0.35)	0.11 (0.10 to 0.11)
Tropical Latin America	28,120 (24,887 to 32,288)	17.89 (15.97 to 20.23)	38,519 (34,603 to 43,240)	17.92 (16.01 to 20.29)	0.01 (−0.01 to 0.03)	9392 (8286 to 10,740)	5.96 (5.30 to 6.73)	12,817 (11,465 to 14,331)	5.96 (5.31 to 6.77)	0.011 (−0.01 to 0.03)
Western Europe	151 (133 to 170)	0.04 (0.04 to 0.05)	159 (142 to 177)	0.04 (0.04 to 0.05)	0.01 (−0.04 to 0.06)	51 (45to 58)	0.01 (0.01 to 0.02)	54 (47 to 60)	0.01 (0.01 to 0.01)	0.01 (−0.04 to 0.06)
Western Sub‐Saharan Africa	5677 (5031 to 6499)	2.81 (2.53 to 3.13)	14,332 (12,626 to 16,445)	2.79 (2.51 to 3.12)	−0.03 (−0.05 to 0.00)	1873 (1657 to 2145)	0.94 (0.84 to 1.05)	4747 (4183 to 5448)	0.92 (0.82 to 1.04)	−0.03 (−0.05 to −0.01)
Eastern Sub‐Saharan Africa	12,422 (11,058 to 14,040)	5.93 (5.37 to 6.58)	24,949 (21,738 to 28,780)	5.43 (4.85 to 6.11)	−0.28 (−0.36 to −0.19)	4184 (3736 to 4676)	2.01 (1.83 to 2.22)	8338 (7176 to 9635)	1.82 (1.62 to 2.05)	−0.34 (−0.38 to −0.29)
High‐income Asia Pacific	390 (343 to 442)	0.23 (0.21 to 0.27)	373 (333 to 414)	0.24 (0.21 to 0.27)	0.03 (−0.01 to 0.08)	130 (114 to 147)	0.08 (0.07 to 0.09)	124 (111 to 138)	0.08 (0.07 to 0.09)	0.03 (−0.01 to 0.08)
High‐income North America	1211 (1074 to 1354)	0.44 (0.38 to 0.49)	1361 (1238 to 1482)	0.40 (0.36 to 0.45)	−0.18 (−0.43 to 0.06)	398 (352 to 448)	0.14 (0.13 to 0.16)	450 (412 to 491)	0.13 (0.12 to 0.15)	−0.17 (−0.41 to 0.07)
North Africa and Middle East	11,632 (10,289 to 13,147)	3.15 (2.83 to 3.52)	19,138 (16,887 to 21,829)	3.04 (2.69 to 3.45)	−0.16 (−0.18 to −0.14)	3855 (3401 to 4320)	1.05 (0.94 to 1.16)	6354 (5560 to 7215)	1.01 (0.89 to 1.14)	−0.14 (−0.21 to −0.08)
Oceania	1679 (1482 to 1945)	23.94 (21.41 to 27.01)	3461 (3074 to 3929)	23.78 (21.29 to 26.56)	−0.02 (−0.03 to −0.01)	556 (486 to 636)	7.96 (7.09 to 8.96)	1146 (1009 to 1300)	7.89 (7.02 to 8.89)	−0.02 (−0.04 to −0.01)
Southern Latin America	115 (100 to 131)	0.23 (0.20 to 0.25)	148 (131 to 168)	0.23 (0.20 to 0.26)	0.04 (0.02 to 0.05)	38 (33 to 44)	0.08 (0.07 to 0.09)	49 (43 to 56)	0.08 (0.07 to 0.09)	0.04 (0.02 to 0.05)
Southern Sub‐Saharan Africa	1693 (1485 to 1945)	3.04 (2.71 to 3.42)	2469 (2191 to 2811)	3.03 (2.70 to 3.44)	0.00 (−0.01 to 0.00)	557 (482 to 639)	1.00 (0.89 to 1.14)	816 (719 to 929)	1.00 (0.89 to 1.13)	−0.005 (−0.01 to 0.002)
World Bank Income										
Low income	14,676 (12,889 to 16,756)	4.56 (4.07 to 5.10)	30,756 (26,958 to 35,280)	4.29 (3.84 to 4.78)	−0.19 (−0.23 to −0.16)	4835 (4241 to 5509)	1.51 (1.35 to 1.69)	10,183 (8868 to 11,674)	1.43 (1.27 to 1.60)	−0.19 (−0.23 to −0.15)
Lower middle income	193,634 (168,029 to 222,117)	8.85 (7.82 to 9.96)	296,414 (260,997 to 336,164)	8.52 (7.52 to 9.59)	−0.14 (−0.22 to −0.05)	63,944 (55,499 to 73,002)	2.93 (2.59 to 3.31)	98,333 (86,026 to 112,146)	2.82 (2.49 to 3.19)	−0.14 (−0.22 to −0.05)
Upper middle income	243,661 (215,326 to 275,660)	11.86 (10.59 to 13.30)	283,319 (253,237 to 315,569)	11.83 (10.55 to 13.28)	−0.01 (−0.02 to 0.00)	80,654 (70,851 to 91,559)	3.92 (3.49 to 4.39)	94,051 (83,752 to 104,895)	3.91 (3.48 to 4.39)	−0.01 (−0.02 to −0.002)
High income	10,368 (9245 to 11,662)	1.06 (0.94 to 1.19)	11,409 (10,267 to 12,643)	1.02 (0.91 to 1.15)	−0.12 (−0.15 to −0.09)	3436 (3053 to 3869)	0.35 (0.31 to 0.39)	3792 (3414 to 4222)	0.34 (0.30 to 0.38)	−0.11 (−0.14 to −0.08)
World Bank Regions										
Sub Saharan Africa	22,268 (19,785 to 25,367)	4.05 (3.66 to 4.49)	47,442 (41,489 to 54,489)	3.80 (3.39 to 4.26)	−0.20 (−0.26 to −0.14)	7170 (6368 to 8086)	1.36 (1.23 to 1.51)	15,773 (13,755 to 18,081)	1.27 (1.13 to 1.43)	−0.22 (−0.29 to −0.15)
Middle East & North Africa	8903 (7918 to 10,045)	3.16 (2.85 to 3.51)	14,443 (12,740 to 16,474)	3.03 (2.68 to 3.44)	−0.16 (−0.18 to −0.14)	2958 (2630 to 3287)	1.05 (0.95 to 1.16)	4804 (4216 to 5461)	1.01 (0.89 to 1.13)	−0.17 (−0.19 to −0.15)
South Asia	114,677 (98,720 to 132,569)	9.30 (8.14 to 10.57)	177,311 (154,435 to 203,118)	9.22 (8.07 to 10.48)	−0.05 (−0.21 to 0.11)	36,927 (31,714 to 42,662)	3.07 (2.68 to 3.50)	58,771 (50,740 to 67,541)	3.05 (2.65 to 3.47)	−0.03 (−0.20 to 0.14)
Europe & Central Asia	10,256 (9121 to 11,559)	1.25 (1.11 to 1.42)	10,339 (9284 to 11,513)	1.25 (1.11 to 1.42)	−0.02 (−0.03 to −0.01)	3407 (3023 to 3847)	0.42 (0.36 to 0.47)	3440 (3082 to 3863)	0.41 (0.36 to 0.47)	−0.02 (−0.02 to −0.01)
East Asia & Pacific	251,475 (221,572 to 284,749)	13.09 (11.67 to 14.70)	295,393 (263,207 to 328,976)	13.39 (11.91 to 15.05)	0.07 (0.07 to 0.08)	83,127 (72,951 to 94,745)	4.32 (3.83 to 4.86)	97,979 (87,266 to 109,589)	4.42 (3.93 to 4.98)	0.07 (0.07 to 0.08)
Latin America & Caribbean	53,458 (47,144 to 60,914)	11.46 (10.22 to 12.93)	75,515 (67,756 to 84,445)	11.94 (10.66 to 13.48)	0.14 (0.11 to 0.17)	17,768 (15,597 to 20,308)	3.81 (3.38 to 4.29)	25,110 (22,369 to 28,166)	3.96 (3.53 to 4.47)	0.14 (0.11 to 0.17)
North America	1217 (1080 to 1360)	0.44 (0.39 to 0.49)	1366 (1243 to 1488)	0.40 (0.37 to 0.45)	−0.19 (−0.43 to 0.06)	400 (354 to 450)	0.14 (0.13 to 0.16)	452 (414 to 493)	0.13 (0.12 to 0.15)	−0.18 (−0.42 to 0.07)
SDI regions										
Low SDI	33,743 (29,603 to 38,490)	6.12 (5.47 to 6.86)	67,501 (58,689 to 77,417)	5.65 (5.03 to 6.35)	−0.28 (−0.39 to −0.17)	11,186 (9780 to 12,669)	2.05 (1.82 to 2.29)	22,395 (19,435 to 25,751)	1.88 (1.66 to 2.12)	−0.30 (−0.41 to −0.18)
Low‐middle SDI	115,493 (100,050 to 132,868)	9.06 (7.99 to 10.19)	166,778 (146,957 to 189,229)	8.52 (7.53 to 9.58)	−0.22 (−0.31 to −0.12)	38,104 (32,961 to 43,651)	3.00 (2.65 to 3.38)	55,283 (48,210 to 63,003)	2.82 (2.49 to 3.18)	−0.22 (−0.31 to −0.13)
Middle SDI	216,433 (190,510 to 245,987)	12.03 (10.73 to 13.47)	268,680 (240,226 to 299,946)	11.41 (10.17 to 12.80)	−0.17 (−0.20 to −0.15)	71,580 (62,743 to 81,570)	3.98 (3.55 to 4.45)	89,186 (79,611 to 99,816)	3.77 (3.36 to 4.22)	−0.17 (−0.20 to −0.15)
High‐middle SDI	83,185 (73,631 to 94,034)	7.76 (6.89 to 8.74)	101,066 (90,202 to 112,619)	8.31 (7.41 to 9.31)	0.22 (0.20 to 0.23)	27,532 (24,205 to 31,387)	2.56 (2.27 to 2.89)	33,558 (29,928 to 37,367)	2.75 (2.44 to 3.09)	0.22 (0.21 to 0.24)
High SDI	13,491 (11,979 to 15,154)	1.59 (1.42 to 1.80)	17,877 (16,077 to 19,879)	1.74 (1.55 to 1.94)	0.27 (0.24 to 0.30)	4468 (3963 to 5044)	0.53 (0.46 to 0.59)	5939 (5328 to 6605)	0.58 (0.51 to 0.65)	0.28 (0.26 to 0.31)

The ASIR and ASPR of scabies showed waves of phase‐wise increases and decreases throughout the 30 years. The largest increase was seen from 2010 to 2015, with a peak occurring in 2015 (Figure S2). The ASIR is approximately three times higher than the ASPR, suggesting that, on average, the duration of the disease is 4 months or a third of a year (since prevalence rate ≅ incidence rate × duration of disease).

### 3.2 Age‐Sex Differences in Scabies

Males had a slightly higher burden of scabies compared to females as their ASIR and ASPR at the global level decreased from 8.43 (95% CI: 7.47 to 9.48) in 1990 to 8.16 (95% CI: 7.25 to 9.14) in 2021 and from 2.80 (95% CI: 2.48 to 3.14) to 2.70 (95% CI: 2.39 to 3.03), respectively (Figure S3). For women, ASIR and ASPR decreased from 8.11 (95% CI: 7.18 to 9.13) in 1990 to 7.94 (95% CI: 7.07 to 8.92) in 2021 and from 2.68 (95% CI: 2.38 to 3.03) in 1990 to 2.63 (95% CI: 2.34 to 2.96) in 2021, respectively.

The gender difference in scabies rates was more pronounced in high and high‐middle SDI regions (Figure S3). Scabies incidence and prevalence counts show an increasing trend until the 5 to 9‐year age group peak, and then gradually decrease (Figure S4A,C). The rates, however, peak at the 2 to 4‐year age group and decrease thereafter, before starting to rise again beyond the 60‐year age mark (Figure S4B,D).

### 3.3 Scabies in Major GBD Regions

Oceania (ASIR_2021_ = 23.78; 95% CI: 21.29 to 26.56; ASPR_2021_ = 7.89; 95% CI: 7.02 to 8.89) had the highest ASPR and ASIR per 100 individuals, followed by Tropical Latin America (ASIR_2021_ = 17.92; 95% CI: 16.01 to 20.29; ASPR_2021_ = 5.96; 95% CI: 5.31 to 6.77) and East Asia (ASIR_2021_ = 14.56; 95% CI: 21.29 to 26.56; ASPR_2021_ = 4.81; 95% CI: 4.28 to 5.40) in both 1990 and 2019 (Table 1). There were no significant reductions in prevalence or incidence of scabies over the last 30 years in these regions. Age‐standardised rates have been the highest in middle SDI regions (ASIR_2021_ = 11.41; 95% CI: 10.17 to 12.80; ASPR_2021_ = 3.77; 95% CI: 3.36 to 4.22), followed by low‐middle and high‐middle SDI. Similarly, upper‐middle income regions showed the highest rates of incidence and prevalence both in 1990 and 2021, followed by lower‐middle‐ and low‐income regions.

### 3.4 National Level Divide in Scabies

**FIGURE 1:**
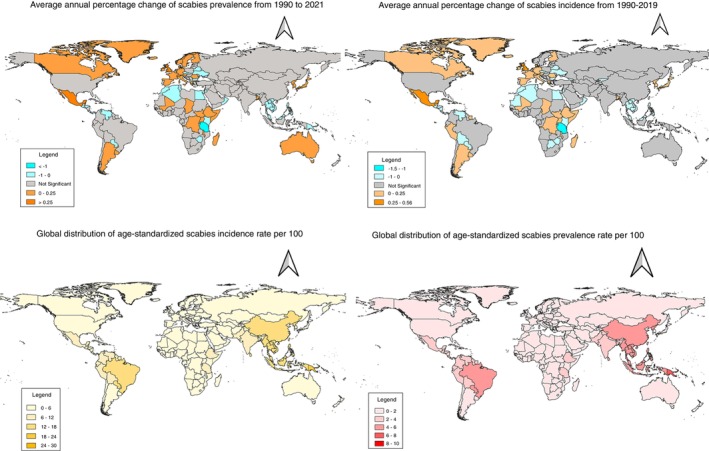
Global spatial distribution of scabies incidence and prevalence and average change from 1990 to 2021.

As of 2021, Fiji had the highest age standardised rates of scabies incidence and prevalence (ASIR = 27.17, 95% CI: 23.45 to 29.37; ASPR = 8.77, 95% CI: 7.75 to 9.83). Fiji was closely followed by other Pacific countries: Papua New Guinea (ASIR = 23.67, 95% CI: 21.16 to 26.49; ASPR = 7.86, 95% CI: 6.98 to 8.83) the Solomon Islands (ASIR = 23.65, 95% CI: 21.10 to 26.54; ASPR = 7.85, 95% CI: 6.98 to 8.83) (Figure 1). This trend has been consistent since 1990.

Over the past 30 years, scabies prevalence and incidence rates have significantly increased in 46 and 47 countries respectively, while a significant decline has occurred in 49 and 51 countries respectively (Table S1). These changes, although statistically significant, were small.

The steepest decline in ASIR from 1990 to 2021 was seen in mostly African countries: Tanzania (AAPC = −1.50; *p* < 0.001), Egypt (AAPC = −0.54; *p <* 0.001), and Kenya (AAPC = −0.29; *p <* 0.001); and some Pacific and Asian countries: Vanuatu (AAPC = −0.05; *p <* 0.001), Lao (AAPC = −0.02; *p <* 0.001) and Vietnam (AAPC = −0.01; *p <* 0.01). In contrast, the largest rise was seen in Mexico (AAPC = 0.56; *p <* 0.001) followed by the United Kingdom (AAPC = 0.27; *p <* 0.001), Romania (AAPC = 0.14; *p <* 0.001) and Italy (AAPC = 0.12; *p =* 0.010).

### 3.5 Temporal Trends in Scabies

**FIGURE 2:**
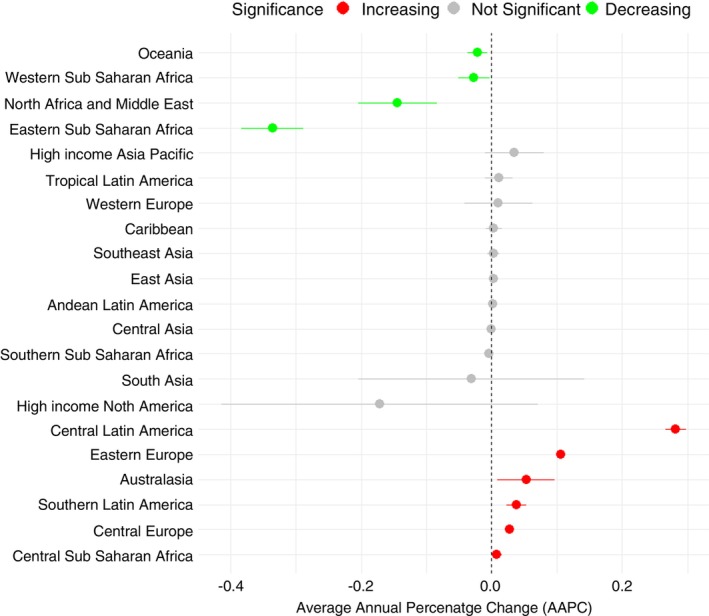
Annual average percentage change of scabies prevalence in the major GBD regions of the world.

The temporal trend of scabies infestation in the major GBD regions is shown in Figure 2, with reduced prevalence in Africa, the Middle East and Oceania and increased prevalence in Europe and the Americas. While a decreasing trend was seen in the sub‐Saharan African regions and Oceania (AAPC = −0.02; 95% CI: −0.03 to 0.00), there was a marked increase in the prevalence rates in the Australasian region (AAPC = 0.05; 95% CI: 0.01 to 0.09) and parts of Europe—Central Europe (AAPC = 0.03; 95% CI: 0.03 to 0.03) and Eastern Europe (AAPC = 0.11, 95% CI: 0.10 to 0.11). The largest increases occurred in Central Latin America with an AAPC of 0.28 (95% CI: 0.27 to 0.30) followed by Eastern Europe and Australasia. The largest decreases were in Eastern Sub‐Saharan Africa with an AAPC of −0.34 (95% CI: −0.29 to −0.34) (Figure 2).

### 3.6 Relationship Between Scabies and SDI

The relationship between scabies and SDI is complex, with an ‘*n*’ shaped relationship between the two variables. Scabies rates have been the highest in middle SDI regions whilst regions on the lower end of the SDI have had scabies rates decline since the 1990s. In contrast, rates have increased in high‐middle and high SDI regions, despite their overall low prevalence.

**FIGURE 3:**
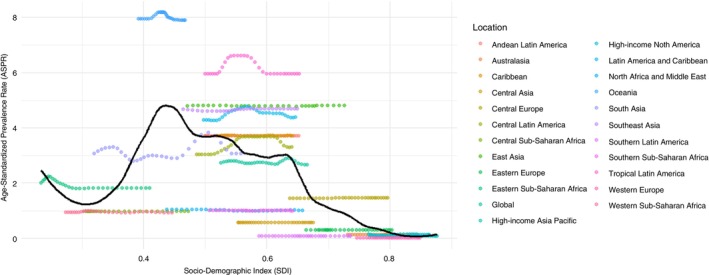
LOWESS curve showing association between SDI and ASPR.

Figure 3 shows an overall static pattern of scabies rates in most other major regions except for Oceania, Tropical Latin America, High‐income North America, and South‐east Asia where rates have increased halfway through the study period before recently returning to their previous level. The moving average (LOWESS) curve shown by the black line indicates a pattern where rates increase from regions with low to middle SDI values before reducing as SDI increases further.

### 3.7 Spatial Clustering

**FIGURE 4:**
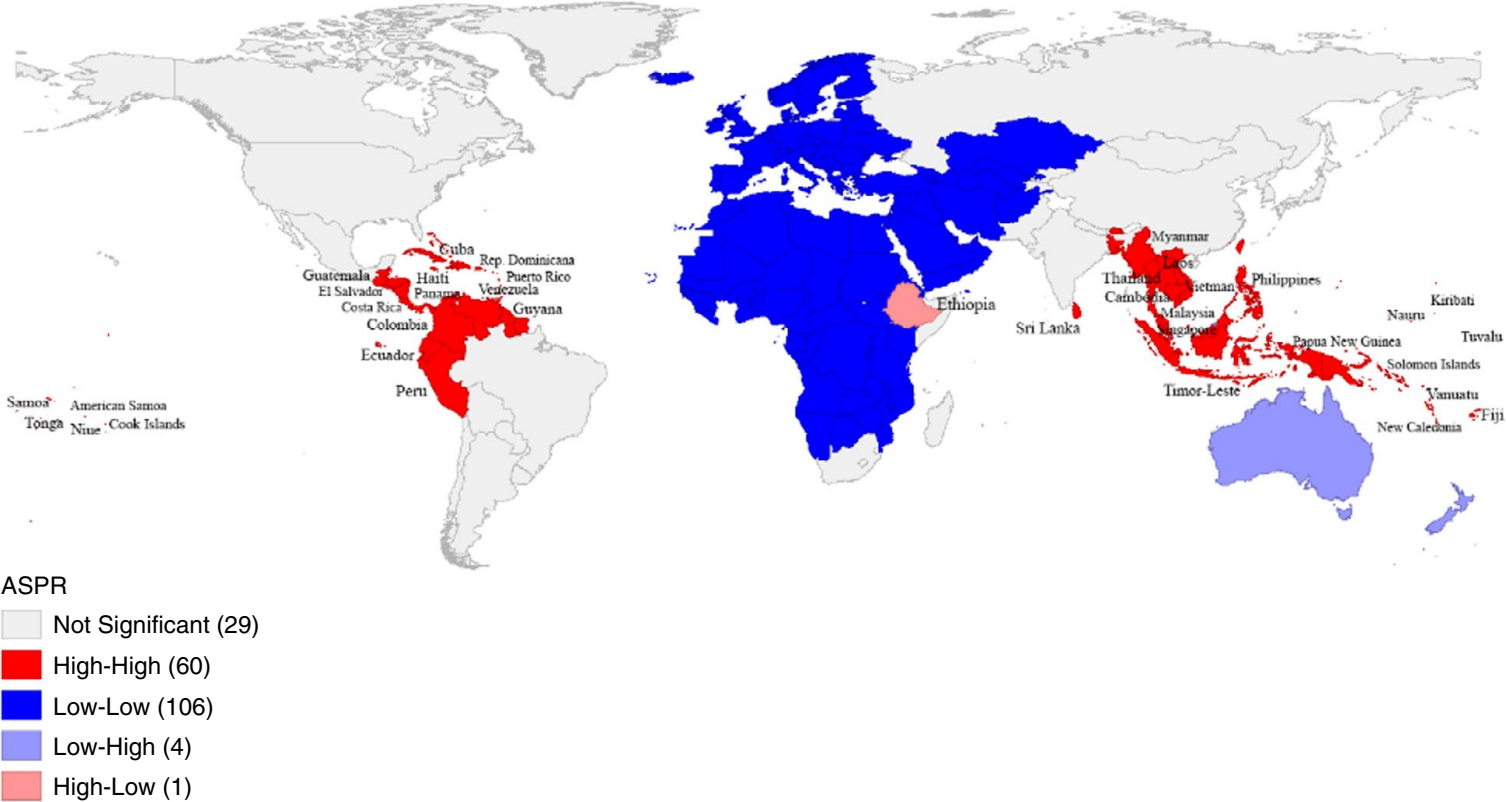
LISA map showing scabies clustering in high and low risk regions.

Moran's *I* for global distribution of scabies rates was 0.78 indicating a high degree of spatial clustering (Figure S5). Scabies ‘hotspots’, or clustering of high prevalence areas, were detected in Tropical Latin America, Southeast Asia and Oceania using the bivariate LISA cluster map (Figure 4).

### 3.8 Risk Factors

As shown in Table S2, latitude and percentage of urban population were negatively associated with scabies frequency in a log‐linear model. While scabies rates decreased on average by 5.52% with each one‐degree change in latitude away from the equator, disease frequency approximately declined by 2.74% with each 1% point increase in urbanisation of the country's population. No significant association was found between population density, percentage of unemployment in the population, life expectancy at birth, gross national income and scabies frequency in the multi‐variable model. A summary of model performance has been shown in Figure S6.

## 4 Discussion

From this analysis of GBD data from 21 regions including 204 countries or territories from 1990 to 2021, we describe a high burden of scabies clustered in Oceania, Tropical Latin America, and Southeast Asia. These findings are consistent with previous GBD estimates [15, 16, 17] and other prevalence studies [18, 19, 20] that have reported high prevalences in countries near the tropics. The highest prevalence of scabies was observed in Pacific countries, for example: Fiji, Papua New Guinea, and the Solomon Islands. Our study has shown periodic fluctuation in global scabies incidence and prevalence, which is very different from the last GBD study that estimated a consistent downward trend of scabies incidence from 1990 to 2017 [15]. This is probably due to the recent change in modelling strategies and the addition of new data sources [9], that may have changed estimates. Consistent with other studies, the data reported here show higher age‐standardised rates of scabies in both young and elderly populations [15].

Our results are consistent with those reported by Lie et al. [17], who also estimated the burden of scabies during a similar time period using the same GBD 2021 dataset. Both studies highlight a high burden of scabies in Oceania, Tropical Latin America, and East Asia, with the highest prevalence observed in Western Pacific Island nations such as Fiji and Tonga. While there are overlapping elements between the two studies in terms of burden estimation and trend analysis, our study extends the scope by identifying statistically significant spatial clusters of high scabies prevalence, understanding the relationship of scabies burden with SDI and, most importantly, the risk factors associated with scabies infestation at the national level. Although estimation of burden and historical rates of the disease are important for surveillance and management, identification of key risk factors is crucial for targeted interventions.

Scabies prevalence and incidence rates have decreased from 1990 to 2021 overall. However, trends show a phase‐wise increase and decrease over the last 30 years. Periodic increases in scabies rates have been seen over 1989 to 1995, 2000 to 2005, and 2010 to 2015. Such cyclical rises in scabies cases are common and have been reported in the past in the United Kingdom, where increases in incident cases every 20 years were reported [21]. Similar trends were also observed in Spain, where hospital admissions due to scabies had a decreasing rate before increasing in the 2014 to 2017 period [20]. While such trends are uncommon in tropical regions like the Pacific Islands, where scabies is endemic, it has been seen in other parts of the world, for example, Europe. Instances of reemergence of scabies cases and occasional outbreaks underline the importance of continued surveillance and updated diagnostic methods even in regions with an otherwise low prevalence like Europe.

One of the key findings of this study is the rising burden of scabies in Europe in recent years, a region that historically reported low prevalences. This finding is supported by anecdotal and empirical evidence from several European countries. Although scabies outbreaks in Europe were previously primarily associated with institutional settings like care homes [22] and educational institutions [23], more recent reports show a rise in national prevalence in certain countries. Between 2013 to 2018, for example, researchers in Norway reported a three‐fold increase in scabies consultations and sales of scabies medications, with a disproportionate burden in young adults in the age range 15 to 29 years [24]. An exponential rise in scabies burden among adolescents and young adults has been reported from Germany where scabies diagnosis increased 9‐fold and treatment failure quadrupled from 2009 to 2018 [25]. Increases in sales of ectoparasiticides have occurred in Spain between 2020 and 2021, where the COVID‐19 lockdowns were suspected to have led to a large‐scale nationwide outbreak of scabies [26]. Italian researchers in Sicily and Lazio have reported large increases in scabies incidence since 2020 [27, 28]. The most recent large‐scale outbreak reported at a national level was in the United Kingdom which led to an acute shortage of medications and was exacerbated by the absence of a national system for tracking scabies cases [29]. A 2022 review of the rising prevalence of scabies in Europe highlights that higher rates are observed among Middle Eastern and African immigrants [20]. The burden of scabies has been increasing, driven in part by worsening living conditions linked to economic crises and the growth in migrant populations [20]. While in the case of institutional outbreaks, MDA for that particular unit must be considered, for a nationwide rise in prevalence, a more comprehensive control policy should be considered, such as improved diagnosis, increased screening and conducting MDA in high‐risk regions.

The burden of scabies has been historically high in the Western Pacific region [18, 19, 30], especially in island countries like Fiji, Vanuatu, Samoa and the Solomon Islands. This has been confirmed by our study, which also showed that the rates of scabies in most of these regions have not decreased in the past 30 years. Scabies prevalence was 16% in Vanuatu in 1989 [31], and has stayed high, being approximately 30% in the most recent prevalence study [32]. A study published in 1999 reporting data from Samoa described a scabies prevalence of 4.9%, thanks to extensive use of ivermectin to eradicate filariasis [33]. However, a recent prevalence study among Samoan schoolchildren reported a prevalence of 14.4% in 2021 [34]. This study suggests that the widespread use of ivermectin during the 1999 study, along with the timing of the current study during the local rainy season—when scabies rates are typically higher—may account for the variation in prevalence estimates. The earliest prevalence study of scabies in Fiji was conducted in 2004, where MDA was conducted in two Fijian villages. At baseline, scabies prevalence was as high as 37.9%, decreasing to 20% after the intervention [35]. Other MDA programs have been successful in Fiji, with an MDA trial conducted in 2019 in the Northern Division of the nation resulting in a substantial reduction of hospitalisations for skin and soft tissue infections in addition to a decline in community prevalence of scabies [36]. Although MDA programs like these have shown promising results in several Pacific, Asian and African nations, prevalence rates of scabies were seen to marginally increase a few years after the MDA program [18]. However, results from such studies show that post‐MDA prevalences were still significantly lower than baseline [37].

While this study provides an overview of scabies prevalence at global and national levels, it is important to acknowledge sub‐national differences. Since the WHO recommends MDA in regions with prevalences higher than 10% [38], it is essential to identify these pockets of high prevalence even in countries with an otherwise low prevalence. For example, although national prevalence of scabies is low overall in Australia, New Zealand, or certain parts of Europe, there have been reported instances where the prevalence is over 10% in certain communities or regions within these countries. For example, scabies is endemic in Aboriginal communities in northern Australia [39]. Scabies prevalence is also high in early childcare centres in socio‐economically disadvantaged areas of Auckland, New Zealand [40]. In Europe, where prevalence of scabies is very low, outbreaks are seen in settings like care homes for the elderly, refugee camps, and hospitals in the United Kingdom [22], Greece [41] and Italy [42]. Since scabies can potentially lead to further complications, these pockets of high prevalence must be taken seriously, with prevalence documented and control programs considered.

The lack of reduction in the prevalence of scabies across the major regions of the world is concerning. Although there has been no alarming increase in scabies rates, the consistent high prevalence in Oceania, Tropical Latin America, and Southeast Asia indicates a lack of progress in disease control in the last three decades. A meta‐analysis of MDA trials has found a relative reduction of scabies prevalence by 79% can be achieved [7]. Although MDA has started in some Pacific nations like Fiji and the Solomon Islands, there is an urgent need to prioritise interventions in all regions with a prevalence > 10%.

There are increasing concerns related to drug resistance reducing the effectiveness of MDA. Substantial resistance of scabies to permethrin has been reported, mainly due to genetic mutations in the mites' voltage‐gate sodium channels and enhanced activity of the detoxifying enzyme glutathione S‐transferase [43]. Concrete evidence for resistance towards ivermectin is yet to emerge. Factors such as prolonged or excessive use of anti‐scabies medications, prolonged treatment, and repeated hospital visits are suspected to have increased drug‐resistant scabies [20].

In addition to drug resistance, another problem is the recurrence of scabies outbreaks within short time intervals. In Italy and Germany, resurgence of scabies cases over short time periods was observed through measurement of repeated prescriptions for scabicides and high numbers of scabies outbreaks in long‐term care facilities [25, 28]. Low adherence to medication occurred in the form of discontinued medications, improper application of scabicides, under‐dosage, especially by young adults, the elderly and psychiatric patients. This has been described as ‘pseudo‐resistance’ and treatment failure [25, 28].

Our study has revealed new potential drivers of scabies prevalence. First, latitude and urbanisation were strongly associated with scabies. No other study has considered the influence of latitude as a risk factor for scabies although it is often referred to as ‘a tropical disease’. Our data are intuitive in a sense that countries farther away from the equator or with more temperate climates have lower scabies rates than warmer, tropical counterparts. Increased urbanisation of populations may lead to poorer living conditions, including overcrowding and poor housing which could explain our finding related to urbanisation. A null association with income levels and scabies differed from previous prevalence studies that found a higher risk of scabies in low‐income households [44, 45]. This may be due to our study's control of a range of confounders. As scabies is likely to be influenced by geographical factors like precipitation, humidity, elevation and temperature [46], further research on understanding the association of these factors is recommended.

To better estimate the true burden of scabies, it is important to reliably and consistently detect cases. Recent developments, such as objective diagnostic tests (qPCR and dPCR) applied to swabs of skin lesions, may help. The International Alliance for Control of Scabies (IACS) consensus criteria also present a robust set of diagnostic criteria which promise to improve sensitivity of scabies detection in a clinical context [47].

Although IACS has been used in some prevalence studies, it is yet to be widely adopted. Our study indicates that global scabies prevalence remains high and progress to reduce scabies prevalence is slow with existing programmes. Scabies control should be further prioritised to accelerate progress to reduce the prevalence of this important neglected tropical disease. Finally, inspiration should be drawn from programmes conducted in Pacific Island nations such as Fiji and the Solomon Islands where nationwide MDA has been used to significantly reduce the prevalence and complications of scabies.

## 5 Threats to Validity

This study has several limitations. Since this study is based on GBD data, it shares the shortcomings of this method for estimating disease burden. The GBD data does not consider differences in prevalence rates due to differences in diagnostic techniques [30]. Although the GBD estimates improve our understanding of the disease burden, especially for a disease like scabies where prevalence information is limited, it also fails to account for likely underreporting.

### 5.1 Issues Related to Underreporting

Underreporting of scabies is a major problem, especially in resource‐poor nations where patients may not seek healthcare due to lack of knowledge about the disease [48], possible stigmatisation, and economic constraints. Similarly, in countries with low prevalence or even in those with high prevalence but possible normalisation of the condition, the index of suspicion for scabies cases is often low, often leading to a failure to seek appropriate healthcare or misdiagnosis. Since scabies cases are often asymptomatic [22], treatment may be initiated for a symptomatic household member while others, despite being infected, may go untreated due to the absence of symptoms. As a result, many cases remain untreated, and only those who seek care are likely to be recorded, leading to considerable undercounting of incidence data.

### 5.2 Misdiagnosis of Scabies

Misdiagnosis of scabies results in an underestimation of the true prevalence of the disease, and also leads to poor treatment efficacy and disease control. Due to secondary bacterial skin infections and resemblance to other skin conditions like insect bites, papulosquamous disorders, non‐infectious inflammatory conditions and infectious dermatoses [49], it is often hard to make an accurate diagnosis of scabies. While traditional methods like visual inspection or skin scrapings have low sensitivity [49, 50], more confirmatory tests like dermoscopy are not accessible in low‐resource countries. However, diagnosis can also be missed even with dermoscopy since the abdomen and eggs of mites are poorly visualised and identifying the mites through the ‘delta wing sign’ requires appropriate training [49].

Scabies detection can be more challenging in certain populations. Among older adults, for example, scabies may be present in atypical forms with complaints of itch being misunderstood for other conditions that occur more commonly in older age groups [51]. This issue may lead to outbreaks and high costs of controlling the disease in long‐term care facilities [52]. The situation can be even more challenging in cases of dementia, where complaints of itch are not made and pruritic marks are frequently absent [53]. Delayed diagnosis can lead to spread of the disease, especially in institutional settings. Such cases are common, as was seen in a study in Taiwan, where 44 episodes of delayed diagnosis of scabies were identified owing to long‐term use of steroids and long duration of hospitalisation [54].

Misdiagnosis of scabies is not just restricted to low‐resource countries, but also prevalent in high‐income countries like New Zealand, where evidence of high prevalence and misdiagnosis of the disease was found in Auckland educational institutions [55]. Scabies misdiagnosis can be a challenge especially due to lack of awareness or experience, especially in areas where the disease is not endemic. Our experience is that scabies is often overlooked, since more sensitive clinical findings, such as crops of papules in an acral distribution, are infrequently interpreted as scabies by primary care physicians [55]. Diagnosis can be even more complicated in the case of crusted scabies as it is often misdiagnosed as psoriasis, irritant dermatitis, or eczema, making it extremely difficult to manage [56, 57].

Such instances of misdiagnosis over prolonged periods of time can lead to severe underreporting of incidence cases in databases like the GBD. The GBD data considers selected prevalence studies from certain regions or communities which are not necessarily reflective of the nationwide prevalence. Given the lack of data from other regions, these studies are used for disease estimation by GBD.

### 5.3 GBD'S Underestimation at the National Level

Since scabies is a neglected tropical disease associated with stigma, has low priority in most nations and is often misdiagnosed, it is extremely difficult to estimate the true prevalence of scabies at the national level. This is why there have been marked differences in prevalence estimates between national surveys in countries like Fiji and the Solomon Islands [58], reporting a prevalence of over 10%, and the GBD estimates. Focused prevalence studies should be conducted in areas with prevalence ranging between 5% and 10% in these GBD estimates. This would help determine whether mass‐drug administration (MDA) to treat scabies should be carried out in such regions in accordance with WHO guidelines.

### 5.4 Study Significance Despite Methodological Shortcomings

The GBD considers imputing data from neighbouring countries and predictive covariates in case of missing data which would likely distort the accuracy of some country's estimates. Given the challenges of data collection and reporting of scabies, it is important to understand that the presented results are modelled estimates synthesising sparse and heterogenous data rather than definitive country‐level estimates. The results presented must therefore be interpreted as indicative patterns to inform hypothesis generation and priority setting rather than definitive confirmatory studies.

The data presented here should not be used as a substitute for local surveys but complements these by providing a global baseline. Rather than relying on exact estimates of disease burden, this study should be considered a reference estimate of scabies incidence and prevalence to help inform advancements in diagnosis, surveillance and primary data collection on scabies, and its prevention and treatment.

## Ethics Statement

This is an observational study that used secondary data that is already published in the open‐source website: https://vizhub.healthdata.org/gbd‐results/. Hence, no ethical approval was required at this stage.

## Conflicts of Interest

The authors declare no conflicts of interest.

## Supporting information


**Data S1:** Supporting Information.

## Data Availability

Data for this study has been extracted from the Global Burden of Disease (GBD) study, 2021: https://vizhub.healthdata.org/gbd‐results/. Codes for statistical analysis of this paper can be found at: https://github.com/Saptorshi1999/Scabies_code_GBD_Study.git.
